# Optimization of Synchropulsed MIG Welding Process Parameters for Welding of AW 5083 Sheets

**DOI:** 10.3390/ma15093078

**Published:** 2022-04-23

**Authors:** Leon Maglić, Dejan Marić, Tomislav Šolić, Ivan Samardžić

**Affiliations:** Mechanical Engineering Faculty, University of Slavonski Brod, 35000 Slavonski Brod, Croatia; tsolic@unisb.hr (T.Š.); isamardzic@unisb.hr (I.S.)

**Keywords:** optimization, MIG synchropulse, geometric properties, AW 5083

## Abstract

Metal inert gas (MIG) welding is one of the processes most commonly used for joining metals, especially for joining aluminum and its alloys. The application of a pulsed current in an electric arc allows better controllability of the molten droplets and the arc transition, which subsequently leads to welds with characteristic flaky joints of better quality. In this paper, the optimization of parameters for welding aluminum alloys using the synchropulse welding process is investigated. By observing the input variables that have the greatest influence on the change in appearance of the welding current characteristics (delta wire feed from 0.1 to 6.0 m/min, frequency F from 0.5 to 3 Hz, duty cycle from 10% to 90%), it is possible to perform an optimization to achieve the desired output values. The output variables of the experiments are defined as insufficient/excessive throat thickness (mm), depth of penetration (mm), and weld width (mm); and for the desired quality of the welded joint the most acceptable range of its values is selected, the numerical optimization implementation. The experiment has shown that the delta wire feed has the greatest effect on the observed properties, while the influence of frequency F and duty cycle is somewhat smaller, but the factors responsible for the observed output properties are still significant. From all this, it is possible to select specific values of these input variables to define the best possible observed properties and to determine the characteristics of the defined mathematical models.

## 1. Introduction

A synchropulse is a function in modern MIG/MAG welding that welds first with high-current pulses and then with the same number of low-current pulses. High-current pulses provide a hot arc (longer arc duration), while low-current pulses allow for the cooling of the weld pool. It alternates the current input of high and low levels according to the determined frequency. Such a process enables greater controllability and precision of the electric arc and reduces spattering [1]. The MIG synchro function enables the optimized welding of sheets of different thicknesses. Laser beam welding and TIG and MIG welding processes, which operate with pulsed energy input, are suitable for joining thin and medium-thickness materials, such as stainless steel, aluminum sheets, and aluminum alloys [1,2].

**Figure 1:**
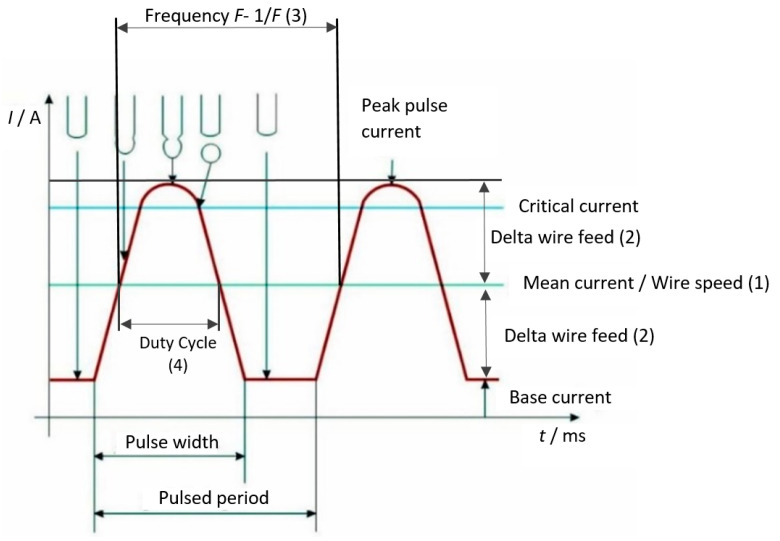
Graphic representation of metal transfer in the pulse current [11].

One of the welding processes that has proven to be effective for welding aluminum and aluminum alloys is the CMT welding process, which allows for less force to be applied and, consequently, causes less deformation and stress in the welded joint [3]. In pulse welding, the current alternates between two extreme values. A low level or low current is called the “background current”, and a high current is called the “pulse current”. When influenced by the pulse current, the transfer of metal within the arc is ideal when it is in the shape of one droplet that is approximately the size of a wire diameter, and it is transferred at low current after each pulsation [4]. The choice of welding parameters has a great influence on the quality of the welded Al alloy T-joint, with the average current and current difference having a significant effect on the macromorphology and microstructure of the welded joint [5]. The measurement and analysis of impulse welding process parameters such as arc temperatures and heat transfer phenomena can be used to predict simulated welding processes [6], which also indicates the need to study influential parameters to improve the process. The authors also compare the results of experimental studies with the results of numerical analysis to determine the relationship and define models that support and accelerate the optimization of the process itself [7,8,9]. The justification for the choice of these input variables for the purpose of studying their interaction also emerges from previously published research that went in a similar direction. There, the importance of welding parameters, including the welding current, wire feed rate, workpiece thickness, speed, and welding geometry, was emphasized. This is also confirmed by the novelty of this work [10]. Pulsed MIG welding operates at two phases of heat input into the material. Within the first phase, the electric arc initially heats the workpiece and removes the oxide layer. In the second phase of heat input, high-current precision pulses melt the filler material and join it with the base. This process results in welded pieces of exceptional quality with minimum spatter. The MIG Synchro welding process can achieve welded joints with flaked shapes. Figure 1 presents the metal transfer in the pulse current by showing the difference between the abovementioned levels of current.

The base current maintains the electric arc, but it does not supply it with enough energy to form a droplet at the tip of the wire electrode. A high current, i.e., pulse current, has a peak amplitude higher than the critical current, which is required to establish the spray arc [12]. Double pulse welding requires precise regulation of the frequency, intermittency, and wire feed speed parameters. Changing these parameters can induce changes in the process characteristics. In particular, the specific wire feed rate can influence the droplet size. By proper adjustment of the frequency parameter, it can be ensured that one droplet is transferred per pulse, and the intermittency affects the final appearance of the welded joint [13]. Double pulse welding changes the intensity of the parameters of the wire feed rate and the current intensity. Moreover, the parameters of low and high current frequencies also change. Therefore, the pulse current is characterized by a double level of parameters referring to the current (base and peak) and the frequency. Along with the parameter of current, there is an increase in voltage, which ultimately results in higher heat input at peak values of current and frequency. Double pulse welding enables a higher energy input than conventional pulse welding. At peak pulse values, the wire and the base material are more melted, while the base pulse levels provide less energy and prevent the burning of the base material [14]. The processes for the optimization of welded joints in the MIG welding process of Al alloys have been shown to be significant for the optimization of parameters (transverse shrinkage, tensile strength, and hardness). Welding current is found to have the greatest influence on these parameters, followed by welding speed and voltage. The Taguchi-based gray relational analysis method was used as the optimization method [15]. The successful application of the response surface method (RMS) in the optimization of the MIG parameters of the welding process, as well as graphical representation of the results and the development of a mathematical model, can be found in other modern studies [16,17,18,19,20].

The article demonstrates the possibility of applying numerical optimization to achieve the best possible properties of the welded joint. Specifically in this example, the geometric characteristics of the weld were used to classify the quality in terms of insufficient/excessive throat thickness, penetration, and weld width. For each of these characteristics, a limit value was set, i.e., the value that is acceptable for the given weld and that is sought to be achieved. Given the above constraints, the values of the input parameters that meet these specifications were determined. The same optimization principle can be applied to other desired numerical quality results of welded joints, which will be the subject of future experimental research.

## 2. Experimental Procedure

Aluminum and aluminum alloys are widely used materials, so both the technology of their processing and the production of new parts of aluminum and aluminum alloys are constantly developing. Aluminum and aluminum alloys have a high specific strength, high processability, anti-erosion properties, increased conductivity, an eco-friendly nature, and recoverability, and are also characterized by good corrosion resistance [21,22]. Having this in mind, the authors conducted research on the application of the MIG synchropulsed welding process for the welding of AlMg4.5Mn0.7 Al alloys [23,24]. The chemical composition of the AlMg4.5Mn0.7 aluminum alloy used in this experiment is given in Table 1. Such types of alloys are usually used in the production of parts and elements for aircraft, ships and railway vehicles [25,26].

The mechanical and physical properties of the alloy used in this experiment are given in Table 2 and Table 3.

Sources of power for the MIG/MAG welding process with the series TPS320i, TPS 400i, TPS 500i, and TPS 600i are completely digital, as there is a microprocessor that controls inverter power sources. The Fronius TPSi 400 (Wels, Austria) was used in this experimental study. The modular design and flexibility of these devices facilitate their operation in any situation. The filler material used in this experiment was an AlMg4.5Mn0.7 alloy, known by its commercial name, AW 5083. Its melting point is approximately 570 °C, and it has very good anticorrosive properties. Applied wire of 1.2 mm diameter was manufactured by Migal Co GmbH (Landau/Isar, Germany). The shielding gas was argon 4.8 I1 (according to the HRN EN ISO 14175:2008 standard). Shielding gas with argon is used for welding aluminum and its alloys, duplex steels, austenitic CrNi steels, other nonferrous metals, gas-sensitive materials, etc.

Defining of Parameters for the Experiment

Based on the analysis of the results of previous studies on welding AW 5083 plates using the synchropulse welding process MIG, the authors found that it is necessary to analyze the interaction of the above parameters and evaluate their influence on the properties of the welded joint, so they defined them as input variables for this experiment. The delta wire feed, the frequency F, and the duty cycle have some influence on the properties of the weld, and these properties are investigated. In this experiment, the obtained results are analyzed to determine the functional dependence in the form of a mathematical model that can be used to further determine the dependence of the tested properties on the input variables. The response surface method (RSM) was used as the experimental design method, i.e., the Central Composite Design (CCD) was chosen. Table 4 provides an overview of the coded values of the factors, while Table 5 shows all experimental runs with an overview of the research results obtained. Table 5 shows the test plan and the arithmetic mean of the responses. The arithmetic mean was calculated for three repeated measurements. The principle of randomization was followed, and 54 samples (three for each experimental condition) were processed in a random experimental order.

The selection of the parameters of the welding machine is limited in view of the values obtained and presented in Table 5. The values of the delta wire feed on the machine vary by ± 0.1 m/min, so exact values could not be obtained. For example, the reading was 1.29592 m/min, but values of 1.3 m/min, 3.1 m/min, and 4.8 m/min were selected; and the frequency parameter varies by ± 0.1 Hz, so values of 2.5 Hz, 1.00 Hz, and 1.8 Hz were selected. Also, the duty cycle parameter can be selected in the range of ±1%, and the selected parameters were 26% and 74%.

Welding is one of the most common processes of joining metals, yet its definition is complex because it involves several scientific disciplines, such as metallurgy, electrical engineering, technical materials, thermodynamics, etc. The multidisciplinarity of the welding process often requires the application of factorial experimental designs, which provide models for output or dependent variables on the basis of input factors or independent variables. In practice, the obtained mathematical model is useful for process optimization and for assuring the better quality of the welded joint. As an example of a welding parameter analysis, a central composite design can be selected by assuming that the generated model will be obtained by a second-degree polynomial. The central composite design belongs to the group of higher-order factorial experiments. It involves a “2*^k^*” factorial design at a high point and a “2*k*” design at the axes and central point, whereas “*k*” represents the number of factors. The number of experimental runs required can be obtained by the following equation [27,28,29,30,31]:

(1) $$N=2^{k}+2\cdot k+n_{0}$$

where *k* is the number of factors and *n*_0_ is the number of repetitions at the central point of the design.

**Figure 2:**
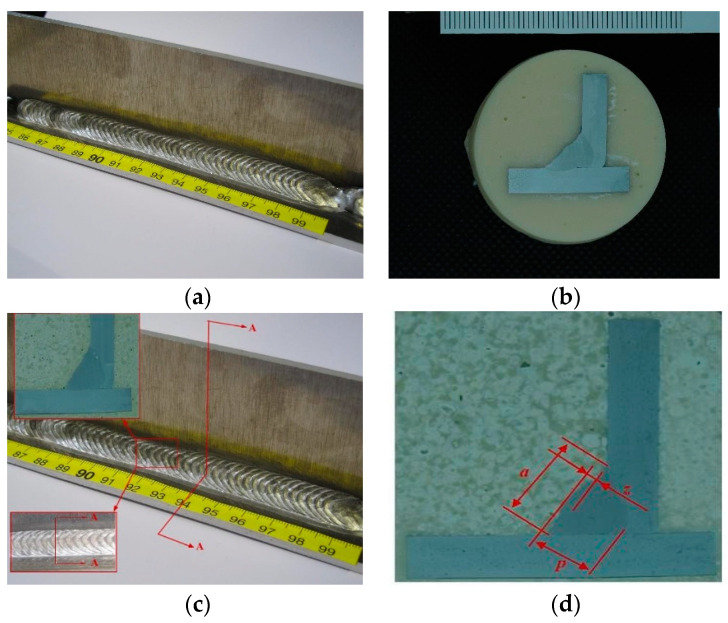
Sample made by welding with the synchropulse welding process: (**a**) Welded joint of the sample No. 17, (**b**) macro sample No. 17, (**c**) cross section of welded joint used for measuring the output variables, (**d**) measuring output variables on thin sections; *a*—weld width (mm); *z*—insufficient/excessive throat thickness (mm); *p*—penetration (mm).

Welding was performed on one side of 18 pairs of plates according to the sequence and parameters specified above. Variable parameters were delta wire feed, frequency, and duty cycle, and constant parameters were welding amperage, welding voltage, welding speed, wire diameter, and arc correction. The angle welds were performed on rectangular specimens with a thickness of 6 mm. Welded specimen No. 17 (see Figure 2a) was taken as a representative specimen since it was welded using nearly ideal parameters. The initial parameters were measured at the cross section of each weld, as shown in Figure 2c. The image obtained by the cross section of the weld was the same for each thin section. Based on the distance measurements on the thin sections, the values of the output variables were obtained, i.e., the values for the insufficient/excessive thickness of the fillet, the penetration, and the weld width, as shown in Figure 2d. The microsection is shown in Figure 2b.

## 3. Analysis of Research Results

In Table 6, samples are marked according to standards of the central composite design. Table 7 shows the maximum and minimum values of the output variables, the mean value, and the standard deviation of the output variables. There is also an optimization model presented for each output variable.

The principle of randomization was respected, i.e., all 18 samples were processed in random order. Sample No. 3 was welded first, while sample No. 5 was welded last. The random order and statistical data analysis were generated in Design Expert software.

### 3.1. Output Variable—Insufficient/Excessive Throat Thickness

Experimental data were used to create a mathematical function that expresses the predicted or average dependence between the response or the output variables and the input factors. This regression model indicates statistical or stochastic dependence. It can be applied in the evaluation, management, and, as in this case, optimization of the process. With reference to the obtained experimental data, it was proven that the linear model was best for different indicators (based on testing the linear model, the linear two-factor interaction model (2FI), quadratic model, and cubic model). The models were tested for the mean squared deviation, for the lack of fit, and for the coefficients of determination, and the obtained data are shown in Table 8.

Table 9 presents the report obtained from Design Expert, referring to the analysis of variance of the selected linear regression model and showing the dependence of insufficient/excessive throat thickness on the input variables.

The coefficient of determination *R*^2^ refers to the share of explained variability (the extent of deviation of the regression *y* from the arithmetic mean) in total variability (the extent of deviation of actual *y* from the arithmetic mean), and is 0.7539 [23]. A higher value of the coefficient of determination does not necessarily mean that the regression model is good. Adding new members to the model will always lead to an increase in *R*^2^, regardless of the statistical significance of a particular member. It is possible that models with a high coefficient of determination actually poorly assess new values or the arithmetic mean of the response. Since *R*^2^ always increases when new members are added to the model, it is necessary to use the adjusted coefficient of determination *R*^2^_adj_, as that value is adjusted to the number of model members in relation to the number of runs, which was 0.7011 in our experiment [23]. Equation (2) presents the regression model of the insufficient/excessive throat thickness dependence on the delta wire feed (factor *A*), frequency (factor *B*), and duty cycle (factor *C*) with coded values for factor levels, while Equation (3) presents the regression model with actual factor values.

(2) z=−0.2378−0.4689A+0.2424B−0.238C

(3) z=0.50701−0.267306 Delta wire feed+0.326124F−0.010006 Duty cycle

**Figure 3:**
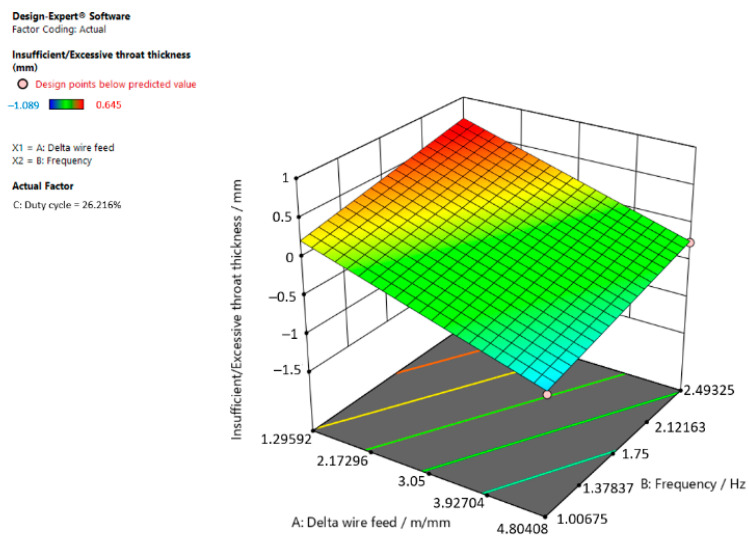
Response surface for the regression model of insufficient/excessive throat thickness at a duty cycle of 26.216%.

**Figure 4:**
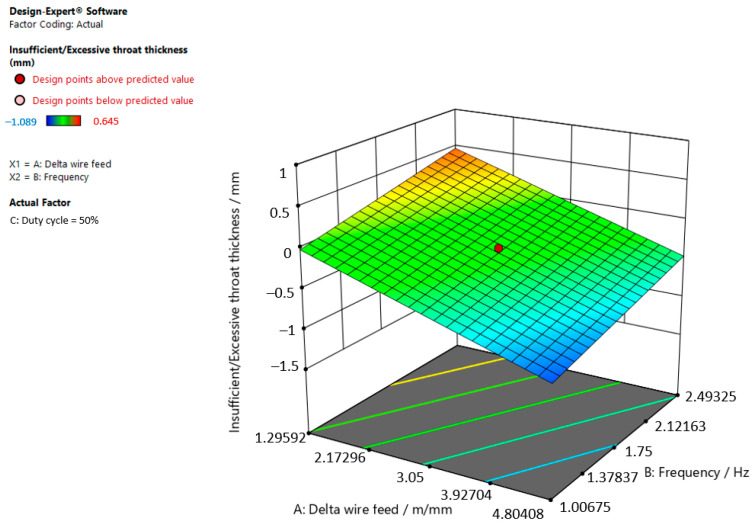
Response surface for the regression model of insufficient/excessive throat thickness at a duty cycle of 50.00%.

**Figure 5:**
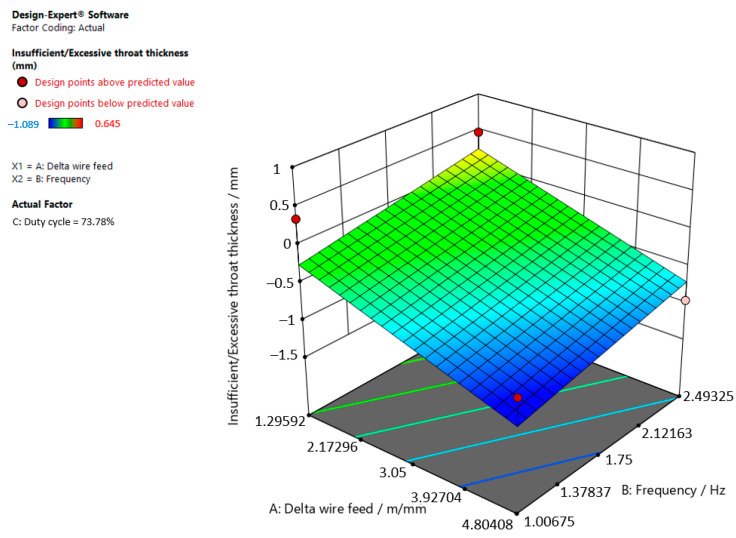
Response surface for the regression model of insufficient/excessive throat thickness at a duty cycle of 73.78%.

Figure 3, Figure 4 and Figure 5 are graphic representations of these models.

Figure 3 shows that insufficient/excessive throat thickness in the weld increases when the delta wire feed parameter decreases and the frequency increases when the duty cycle is 26.216%.

Figure 4 shows that the insufficient/excessive throat thickness in the weld increases when the delta wire feed parameter decreases and the frequency increases when the duty cycle is 50.00%.

Figure 5 shows that the insufficient/excessive throat thickness in the weld increases when the delta wire feed parameter decreases and the frequency increases when the duty cycle is 73.78%.

### 3.2. Output Variable—Weld Penetration

The linear model proved to be the best for the experimental data. The models were tested for the mean squared deviation, lack of fit, and coefficients of determination, and the obtained data are given in Table 10.

Table 11 presents the report obtained from Design Expert referring to the analysis of variance of the selected linear regression model that is used to describe the dependence of weld penetration on the input factors.

The coefficient of determination *R*^2^ refers to the share of explained variability in total variability, which in this case is 0.7689. The adjusted coefficient of determination *R*^2^_adj_ is adjusted to the number of model members in relation to the number of runs, and is 0.7194. Equation (4) shows the regression model for the dependence of weld penetration on the delta wire feed (factor *A*), frequency (factor *B*), and duty cycle (factor *C*) with coded values for factor levels, while Equation (5) presents the regression model with actual factor values.

(4) p=6.8−0.6869A+0.1066B+1.22C

(5) p=5.17525−0.391577 Delta wire feed+0.143467F+0.051393 Duty cycle

**Figure 6:**
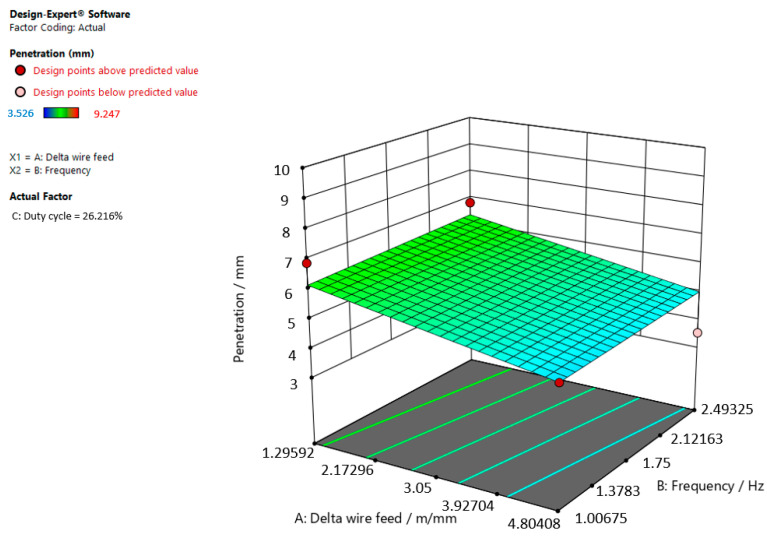
Response surface for the regression model of weld penetration at a duty cycle of 26.216%.

**Figure 7:**
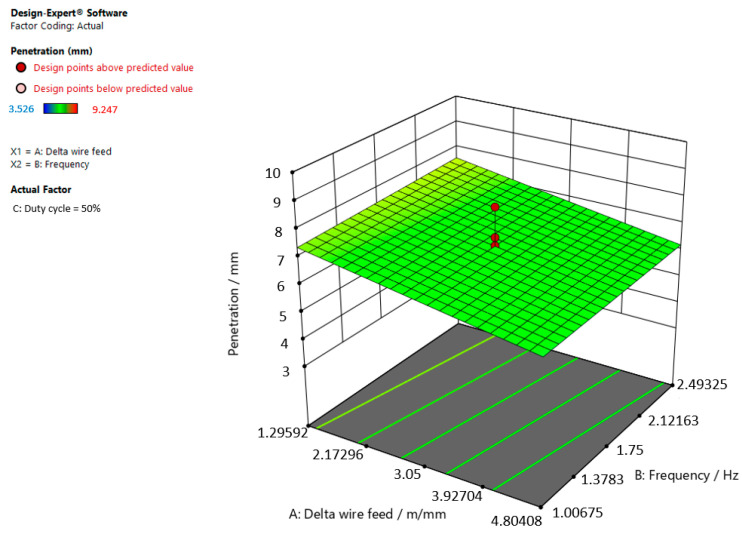
Response surface for the regression model of weld penetration at a duty cycle of 50.00%.

**Figure 8:**
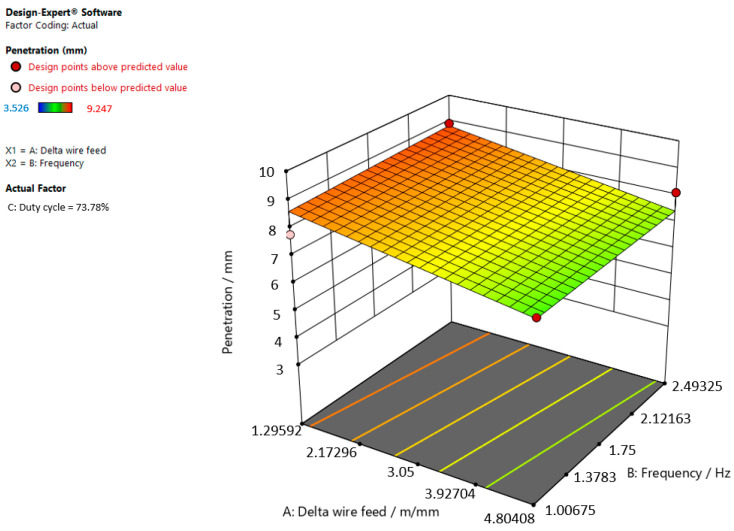
Response surface for the regression model of weld penetration at a duty cycle of 73.78%.

Figure 6, Figure 7 and Figure 8 refer to the graphic presentations of the models.

Figure 6 shows that the superelevation in the weld increases and the delta wire feed parameter decreases when the duty cycle is 26.216%.

Figure 7 shows that the superelevation in the weld increases and the delta wire feed parameter decreases when the duty cycle is 50.00%.

Figure 8 shows that the superelevation in the weld increases and the delta wire feed parameter decreases when the duty cycle is 73.78%.

### 3.3. Output Variable—Weld Width

The linear model again proved to be the best for the experimental data. The models were tested for the mean squared deviation, lack of fit, and coefficients of determination, and the obtained data are shown in Table 12.

Table 13 refers to the report obtained from Design Expert, referring to the analysis of variance of the selected linear regression model for presentation of the weld width dependence on the input factors.

The coefficient of determination *R*^2^ is 0.7475. The adjusted coefficient of determination *R*^2^_adj_, adjusted to the number of model members in relation to the number of runs, is 0.6932. Equation (6) shows the regression model for the dependence of weld width on the delta wire feed (factor *A*), frequency (factor *B*), and duty cycle (factor *C*) with coded values for factor levels, while Equation (7) presents the regression model with actual factor values.

(6) a=10.44+0.6338A−0.24B+1.17C

(7) a=7.45033−0.361312 Delta wire feed+0.322915F+0.049053 Duty cycle

**Figure 9:**
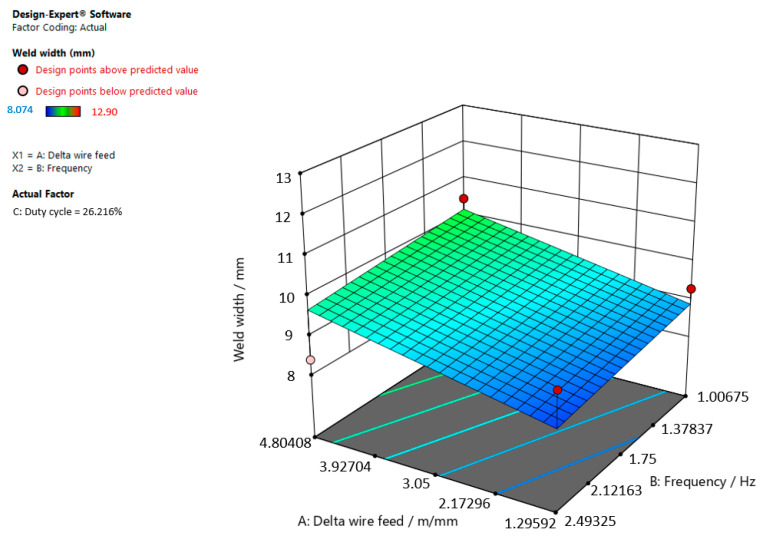
Response surface for the regression model of weld width at a duty cycle of 26.216%.

**Figure 10:**
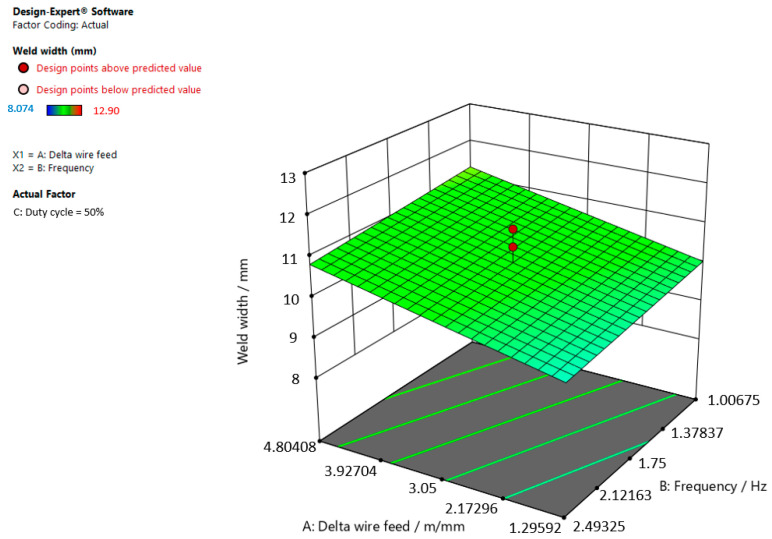
Response surface for the regression model of weld width at a duty cycle of 50.00%.

**Figure 11:**
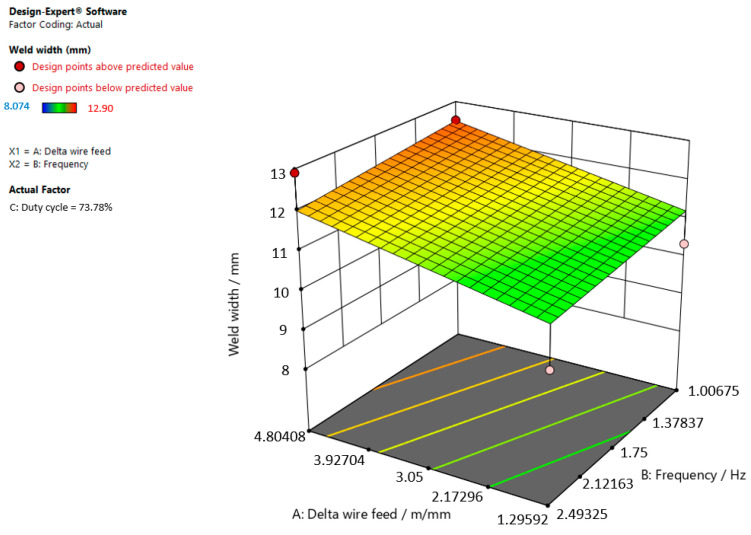
Response surface for the regression model of weld width at a duty cycle of 73.78%.

Figure 9, Figure 10 and Figure 11 give graphic representations of these models.

Figure 9 shows that the weld width increases, the delta wire feed parameter increases, and the frequency slightly decreases when the duty cycle is 26.216%.

Figure 10 shows that the weld width increases, the delta wire feed parameter increases, and the frequency slightly decreases when the duty cycle is 50.00%.

Figure 11 shows that the weld width increases, the delta wire feed parameter increases, and the frequency slightly decreases when the duty cycle is 73.78%.

Based on the research results, the parameters could be optimized by selecting the values of the input variables that provided the desired characteristics. Table 14 gives an overview of the limits set for the above optimization. Ranges were given within which the input variables and the desired property to be achieved by the optimization were kept. The goal was to achieve a throat thickness that was too small/excessive with a target value of 0, a weld penetration that was as large as possible, and a weld width with a target value of 10. In selecting the optimal values for the input parameters, an importance factor was added for each service in addition to the constraints listed in Table 14. By degree of importance, penetration was determined to be the most important factor, while insufficient/excessive throat thickness was determined to be the least important factor.

Given the defined constraints, Table 15 shows the solutions for some of the best combinations of input variables.

**Figure 12:**
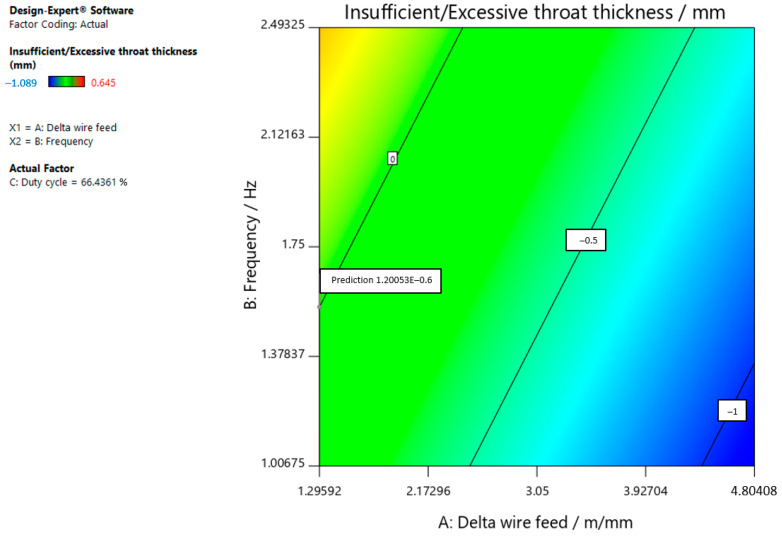
Optimal parameters for achieving the insufficient/excessive throat thickness with a target value of 0.

**Figure 13:**
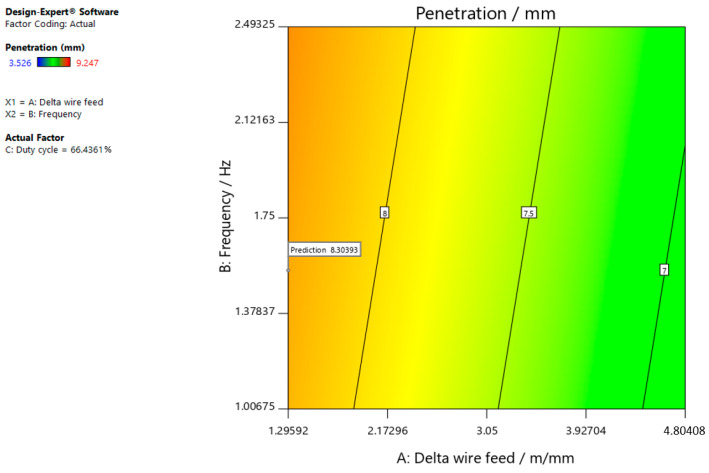
Optimal parameters for achieving as much penetration as possible.

**Figure 14:**
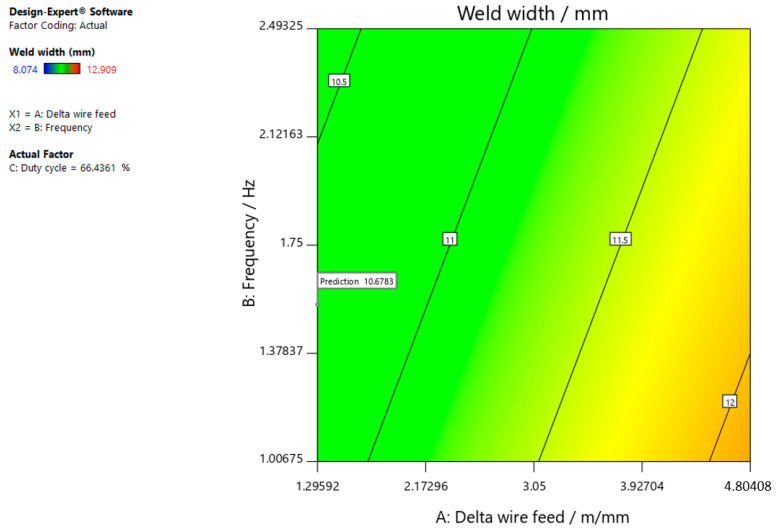
Optimal parameters for achieving the weld width with a target value of 10.

The optimal parameters (number 1) that resulted in insufficient/excessive neck thickness with a target value of 0, maximum penetration, and weld width with a target value of 10 are shown in Figure 12, Figure 13 and Figure 14. To obtain the desired characteristics, it is best to select the following values for the input parameters: delta wire feed = 1.296 m/min; frequency = 1.546 Hz, and duty cycle = 66.436%, which can also be seen in the following figures.

## 4. Conclusions

The research objective was to test the influence of the input parameters of the frequency, duty cycle, and change in the welding wire feed rate on the output variables of the insufficient/excessive throat thickness, weld penetration, and weld width to determine the optimal parameters for the welding of steel. The experiment was performed by welding samples of AlMg4.5Mn0.7 aluminum alloy, also known as AW 5083, with argon as the shielding gas. Analysis of the experimental results obtained by testing the output variables showed that sample No. 11 achieved the lowest value of insufficient/excessive throat thickness, with a convexity of 1.089 mm. The highest insufficient/excessive throat thickness of 0.645 mm was measured on the welded joint of sample No. 13. The lowest value of the output variable of weld penetration was on sample No. 4, which was 3.526 mm. In contrast, the highest value of weld penetration was on sample No. 14, which was 9.247 mm. The third observed output variable of weld width achieved the lowest value on sample No. 13 (8.074 mm) and the highest value on sample No. 8 (12.909 mm). It can be concluded that sample No. 13 had the highest value of reinforcement but also the lowest value for the weld width. The values of other outputs for each sample are given in Table 12.

An analysis of the experimental results proved that the AW 5083 aluminum alloy exhibited optimal welding parameters with samples No. 15–18, which was also clearly visible upon a visual inspection of the samples. This paper has elaborated mathematical models to confirm that they can serve as a good basis for prediction and analysis of the influence that input parameters have on the analyzed output variables. Moreover, the mathematical models created can be used for simultaneous optimization of different output variables, i.e., of the response, to ensure better and easier welding process control.

## Figures and Tables

**Table 1:** Chemical composition of AlMg4.5Mn0.7 alloy.

Chemical Element	Content/%
Mn	0.40–1.00
Fe	0.40 max
Cu	0.10 max
Mg	4.00–4.90
Si	0.0–0.40
Zn	0.0–0.10
Cr	0.05–0.25
Ti	0.05–0.25
Other alloy elements	0.0–0.15
Al	Balanced

**Table 2:** Mechanical properties of AlMg4.5Mn0.7 alloy.

Mechanical Property	Value
Proof strength *R*_p0.__2_ (Mpa)	110
Tensile strength *R*_m_ (Mpa)	270
Elongation *A* (%)	12
Elongation *A*_50_ (%)	10
Brinell hardness (*HB*)	75

**Table 3:** Physical properties of AlMg4.5Mn0.7 alloy.

Physical Property	Value
Density (g/cm^3^)	265
Melting point (°C)	570
Thermal dilatation (1/K)	25 × 10^−6^
Modulus of elasticity (*E*/Gpa)	72
Thermal conductivity (*W*/mK)	121
Electrical resistance (Ωm)	0.058 × 10^−6^

**Table 4:** Coded values of the experiment design.

Coded Values	Factor 1—Delta Wire Feed (m/min)	Factor 2—Frequency (Hz)	Factor 3—Duty Cycle (%)
−1.68179	0.1	0.5	10
−1	1.29592	1.00675	26.2159
0	3.05	1.75	50
1	4.80408	2.49325	73.7841
1.68179	6	3	90

**Table 5:** Overview of values referring to input variables as determined by Design Expert software (Minneapolis, MN, USA).

Ordinal Number of the Run	Sample Number	Parameter 1	Parameter 2	Parameter 3
		*A*: Delta Wire Feed (m/min)	*B*: Frequency (Hz)	*C*: Duty Cycle (%)
3	1	1.29592	2.49325	26.2159
12	2	3.05	3	50
6	3	4.80408	1.00675	73.7841
2	4	4.80408	1.00675	26.2159
13	5	3.05	1.75	10
11	6	3.05	0.5	50
16	7	3.05	1.75	50
8	8	4.80408	2.49325	73.7841
18	9	3.05	1.75	50
10	10	6	1.75	50
14	11	3.05	1.75	90
17	12	3.05	1.75	50
4	13	4.80408	2.49325	26.2159
7	14	1.29592	2.49325	73.7841
9	15	0.1	1.75	50
15	16	3.05	1.75	50
1	17	1.29592	1.00675	26.2159
5	18	1.29592	1.00675	73.7841

**Table 6:** Obtained output values.

Sample Number	Parameter 1	Parameter 2	Parameter 3	Output 1	Output 2	Output 3
	*A*: Delta Wire Feed (m/min)	*B*: Frequency (Hz)	*C*: Duty Cycle (%)	Insufficient/Excessive Throat Thickness (mm)	Penetration (mm)	Weld Width (mm)
1	6	1.75	50	−0.943	5.492	10.957
2	3.05	1.75	50	−0.258	6.188	10.113
3	4.80408	1.00675	26.2159	−0.753	4.786	10.443
4	3.05	0.5	50	−1.089	6.176	11.341
5	4.80408	2.49325	73.7841	−0.957	8.126	12.909
6	1.29592	1.00675	73.7841	0.347	7.791	10.350
7	4.80408	2.49325	26.2159	−0.244	3.526	8.386
8	3.05	1.75	50	−0.176	8.224	10.839
9	1.29592	2.49325	26.2159	0.515	6.828	9.324
10	3.05	1.75	50	−0.372	6.821	9.738
11	3.05	1.75	10	0.645	5.301	8.074
12	4.80408	1.00675	73.7841	−0.82	7.267	12.497
13	3.05	3	50	0.409	6.656	10.762
14	3.05	1.75	90	−0.873	9.247	12.809
15	1.29592	1.00675	26.2159	0.218	6.893	9.288
16	1.29592	2.49325	73.7841	0.469	8.906	9.655
17	0.1	1.75	50	0.294	7.078	9.151
18	3.05	1.75	50	−0.693	7.124	11.282

**Table 7:** Characteristic values of output variables for selected models.

Output Variable	The Lowest Value	The Highest Value	Mean Value	Standard Deviation	Model
Insufficient/Excessive throat thickness (mm)	−1.089	0.645	−0.2378	0.5977	Linear
Penetration (mm)	3.526	9.247	6.80	1.44	Linear
Weld width (mm)	8.074	12.909	10.44	1.40	Linear

**Table 8:** Simulation of four models in the design expert insufficient/excessive throat thickness.

Model	Mean Squared Deviation (*p*-Value)	Lack of Fit (*p*-Value)	Adjusted Coefficient of Determination	Predicted Coefficient of Determination
Linear	0.0002	0.2593	0.7011	0.5675
2FI	0.6934	0.2124	0.6648	0.1350
Quadratic	0.7646	0.1474	0.5977	−0.2975
Cubic	0.0700	0.4910	0.8699	−0.1901

**Table 9:** Analysis of variance for the regression model–insufficient/excessive throat thickness.

Source of Variance	Sum of Squares	Degrees of Freedom	Mean Squared Deviation	*F* Value	*p*-Value
Model	4.58	3	1.53	14.29	0.0002
*A*—Delta wire feed (m/min)	3.00	1	3.00	28.12	0.0001
*B*—Frequency (Hz)	0.8024	1	0.8024	7.52	0.0159
*C*—Duty cycle (%)	0.7734	1	0.7734	7.24	0.0175
Residual	1.49	14	0.1068		
Lack of fit	1.34	11	0.1218	2.37	0.2593
Pure error	0.1544	3	0.0515		
Total	6.07	17			

**Table 10:** Simulation of the four models in Design Expert penetration.

Model	Mean Squared Deviation (*p*-Value)	Lack of Fit (*p*-Value)	Adjusted Coefficient of Determination	Predicted Coefficient of Determination
Linear	<0.0001	0.6887	0.7194	0.6187
2FI	0.0703	0.8771	0.8070	0.7636
Quadratic	0.2928	0.9526	0.8291	0.7446
Cubic	0.8859	0.7980	0.7302	0.5346

**Table 11:** Analysis of variance for the regression model–weld penetration.

Source of Variance	Sum of Squares	Degrees of Freedom	Mean Squared Deviation	*F* Value	*p*-Value
Model	27.00	3	9.00	15.53	<0.0001
*A*—Delta wire feed (m/min)	6.44	1	6.44	11.11	0.0049
*B*—Frequency (Hz)	0.1553	1	0.1553	0.2679	0.6128
*C*—Duty cycle (%)	20.40	1	20.40	35.20	<0.0001
Residual	8.12	14	0.5797		
Lack of fit	5.94	11	0.5403	0.7458	0.6887
Pure error	2.17	3	0.7244		
Total	35.12	17			

**Table 12:** Simulation of four models in the design expert weld width.

Model	Mean Squared Deviation (*p*-Value)	Lack of Fit (*p*-Value)	Adjusted Coefficient of Determination	Predicted Coefficient of Determination
Linear	0.0002	0.4649	0.6932	0.5564
2FI	0.0706	0.6479	0.7888	0.4711
Quadratic	0.5366	0.5945	0.7755	0.3917
Cubic	0.4072	0.7083	0.8034	0.3768

**Table 13:** Analysis of variance for the regression model–weld width.

Source of Variance	Sum of Squares	Degrees of Freedom	Mean Squared Deviation	*F* Value	*p*-Value
Model	24.86	3	8.29	13.81	0.0002
*A*—Delta wire feed (m/min)	5.49	1	5.49	9.14	0.0091
*B*—Frequency (Hz)	0.7867	1	0.7867	1.31	0.2715
*C*—Duty cycle (%)	18.59	1	18.59	30.97	<0.0001
Residual	8.40	14	0.6003		
Lack of fit	6.95	11	0.6316	1.30	0.4649
Pure error	1.46	3	0.4856		
Total	33.27	17			

**Table 14:** Defined limitations for optimization of parameters.

Name	Goal	Lower Limit	Upper Limit	Importance
*A*: Delta wire feed	is in range	1.29592	4.80408	3
*B*: Frequency	is in range	1.00675	2.49325	3
*C*: Duty cycle	is in range	26.2159	73.7841	3
Insufficient/Excessive throat thickness	is target = 0	−1.089	0.645	1
Penetration	maximize	3.526	9.247	3
Weld width	is target = 10	8.074	12.909	2

**Table 15:** Solution for five combinations of input variables.

Number	Delta Wire Feed	Frequency	Duty Cycle	Insufficient/Excessive Throat Thickness (mm)	Penetration (mm)	Weld Width (mm)
1	1.296	1.546	66.436	0.000	8.304	10.678
2	1.296	1.540	66.240	0.000	8.293	10.671
3	1.296	1.554	66.692	0.000	8.318	10.688
4	1.296	1.533	66.026	0.000	8.281	10.662
5	1.296	1.558	66.817	0.000	8.325	10.693
6	1.296	1.527	65.806	0.000	8.269	10.654
7	1.296	1.566	67.093	0.000	8.341	10.704
8	1.296	1.574	67.341	0.000	8.354	10.714
9	1.296	1.513	65.361	0.000	8.244	10.636
10	1.296	1.580	67.554	0.000	8.366	10.722
11	1.296	1.503	65.042	0.000	8.226	10.624
12	1.296	1.495	64.782	0.000	8.212	10.614
13	1.299	1.509	65.159	0.000	8.232	10.629
14	1.302	1.552	66.477	0.000	8.305	10.680
15	1.296	1.614	68.665	0.000	8.428	10.766
16	1.296	1.623	68.947	0.000	8.444	10.777
17	1.296	1.614	67.962	0.007	8.392	10.731
18	1.296	1.673	70.595	0.000	8.536	10.841
19	1.332	1.549	65.556	0.000	8.245	10.647
20	1.296	1.742	72.815	0.000	8.660	10.928
21	1.296	1.314	58.871	0.000	7.882	10.382
22	1.296	1.971	73.784	0.065	8.743	10.901
23	1.296	1.244	57.374	0.008	7.795	10.331
24	1.296	2.024	72.296	0.097	8.674	10.811
25	1.296	2.028	73.780	0.084	8.751	10.883
26	1.296	1.161	53.885	0.000	7.604	10.187
27	1.296	1.875	64.340	0.128	8.243	10.469
28	1.296	1.078	51.205	0.000	7.454	10.082
29	1.296	1.050	51.196	0.009	7.450	10.091
30	1.296	1.028	49.558	0.000	7.362	10.018
31	1.670	2.258	73.784	0.059	8.637	10.944

## Data Availability

The data presented in this study are available from the corresponding author, upon reasonable request.
